# The Etiology, Diagnosis and Treatment of Gall Stones

**Published:** 1907-07

**Authors:** Willis Jones

**Affiliations:** Atlanta, Ga.


### THE ETIOLOGY, DIAGNOSIS AND TREATMENT OF GALL STONES.

By Willis Jones, Atlanta, Ga.

Some idea of the frequency of this condition may be had from the following statistics: In 9,000 autopsies Naunyn found stones in one in every thirty individuals before thirty years of age, and in one in every six after sixty years of age. Bollinger found stones in 2.7 per cent. between the years of fifteen and thirty, in 5.9 per cent. between the years of thirty and sixty and in 15.2 per cent. after sixty years of age. Working along these same lines Schrœder found stones present in 12.5 per cent. of all his autopsies. Still has reported 20 cases in children, 10 of which were infants. Kerh, in 360 operations on the gall bladder and its passages, found stones present in 307 of the 360 cases. Striking an average of the above figures, we find that the condition is present in from 10 to 12 per cent. of all individuals and occurring about four times as often in the female as the male. So we learn that probably no other surgical condition in the abdominal cavity occurs quite so frequently.

No one etiological factor, as we will see further on, can be held accountable for such a prevalent condition, and if we go searching for the cause we will have to consider the union of several factors as essential to their formation. From the prevalence of the condition among those who have passed the middle line of life, and since statistics show us that gall stones occur about four times as often in the female as the male, we must look upon old age, and especially in women, for one of our chief factors in their production. In this class of patients we are confronted with arterio-sclerosis, retarded metabolism and usually sedentary habits; and in women more frequently than men, because the bile passages are so interfered with by pregnancy, the mode of dress and tight lacing, that stagnation results and this stagnation or bile stasis predisposes to precipitation of its solid constituents.

Bacterial infection has proven to be a promising field, but is more likely to be secondary to some form of obstruction, for the free flow of bile through its passages has a tendency to wash out any infecting agents, and furthermore, human bile per se is sterile, and under normal conditions, does not furnish proper food for the growth and propagation of micro-organisms. The bacillis typhosus and the bacillus coli communis are frequently a nuecleus of gall stones and also found in the gall bladder. There are two channels through which they can reach the gall bladder, one by the portal circulation and the other through the common duct from the duodenum. Another fact in favor of bacterial origin is that over 20 per cent. of cases operated on for gall stones give a previous history of having had typhoid fever. Likewise any of the acute infectious diseases may predispose by leaving the gall bladder catarrhally diseased.

As the disease occurs among all classes and in all countries, too much stress must not be laid upon climate and diet. The investigations of Beadles, Gorstell and Snell, show that confinement and sedentary habits have their influence, for their autopsies upon a large number of insane individuals showed stones present in 21.2 per cent. of cases, substantiating the views of some that physical repose, more than mental activity, is responsibe for their development.

On analysis, gall stones are found to be composed of cholesterine alone, biliruben-calcium alone, or varying proportions of both, with cholesterine usually predominating. Cholesterine, the physiologic chemist tells us, is present in bile in small quantities at all times, and increased by any inflammatory change in the bile passages. Its origin is thought to be due to retrograde matamorphosis of the epithelial cell protoplasm. Biliuben-calcium, the other important constituent of gall stones, has the following origin: biliruben, a sulphur containing albuminoid derivitive, is a normal constituent of bile, which is secreted by the healthy functionating liver cells and is held in solution by the bile and is not a product of the degeneration as is cholesterine. During certain pathological disturbances, it may be precipitated by a calcium basis forming biliruben-calcium which is insoluble in bile.

In 1892 Naunyn and his co-workers gave the following views concerning the origin of gall stones: (1.) He thinks that all the solids in body oils are independent of body conditions; that cholesterine is a product of the bile passages, and as such, is not influenced by any diathetic or non-local condition. (2d.) That bacteria enter the bile system, set up an inflammation, catarrhal in type, which blocks the free flow of bile, and is accompanied by each increased production of cholesterine from the inflamed mucous membrane of the gall bladder and by an exudation of albuminous substances. In this bacterial inflammation, all the elements of the gall stones are present, and by a process of precipitation and infiltration, stones develop absolutely independent of the general body conditions.

McArthur thinks that all gall stones do not originate in the gall bladder; that the origin of cholesterine stones is probably in the gall bladder with the subsequent growth within the bladder or its ducts where they may find their way and lodge; that biliruben-calcium is the constituent of the smaller intra-hepatic duct stone.

In summing up the etiological factors in the gall stone formations the following conclusions seem apparent: (1st.) That the chief constituents of gall stones, cholesterine and biliruben-calcium, are produced by subacute inflammatory changes in the membrana mucosa of the gall bladder, resulting in a disintegration of its epithelium and an increased production of mucus. (2d.) That any agent capable of disturbing the flow of bile through its channels favors the introduction of micro-organisms, which in turn pre-disposes to catarrhal inflammation.

The gall bladder, located as it is in the right upper quadrant of the abdominal cavity, often presents to the internist as well as to the surgeon, symptoms which are perplexing as well as puzzling, for here we are called upon to differentiate between pathological lesions arising not only in the gall bladder and its ducts, but as well in the pyloric end of the stomach, duodenum, head of pancreas, kidney and last but not least of importance, the vermiform appendix. The diagnosis of the so-called classical cases of gall stone diseases is fairly easy and comparatively certain, but where the symptoms are obscure and ambiguous, a reasonable doubt must always exist, or embarrassing mistakes will be made. The typical symptoms of biliary colic and of obstruction of the common duct are so readily recognized that only mention will be made of the diagnosis of the attypical types. For the majority of us with a given history of a severe attack, or of repeated attacks of paroxysmal pain in the hepatic region associated with or without jaundice, in a middle aged individual, particularly so if it be a woman, will think of gall stone disease. In a large number of cases our diagnosis can only rest on a suspicion, and it is concerning this variety that I wish to say most.

If we are to meet the diagnosis of gall bladder pathology consistently, we have many long held fallacies or misinterpretations to as well as many truths to remember. Were neuralgias of the stomach, gastralgia, cardalgias and acute indigestion forever buried, there would be made better diagnosis, and greater number of relieved and rejuvenated patients to rejoice over the graves of these buried bugbears. Granting that these conditions may occur as independent realities, they will usually be found associated with some lesions such as cicatrix, ulcer, cancer or hyper-secretion of stomach or duodenum, with a reasonable history leading directly to such condition.

In the Mayo clinics more than 90 per cent. of the so-called neuralgias of the stomach have proven to be gall bladder trouble, and the remaining 10 per cent. due chiefly to diseases of the duodenum and appendix. It is in those patients who give histories of repeated attacks of gastralgia of acute indigestion brought on usually after taking food, that we have to study cautiously and continuously to be able to exclude one by one of the various possibilities of disease in the organs of the upper right quadrant, before definite conclusion can be drawn. Such patients are usually of a hysterical temperament, have consulted various specialists and received many and various diagnosis; and ofttimes before you have had time to study exhaustingly, they have decided to go to some one else for more and different advice. To the majority of such sufferers the diagnosis of some form of neuralgia has usually been made, and this appeals to them much more strongly than any diagnosis which may mean surgical interference. A great number are inclined to believe in that diagnosis, the treatment for which is most pleasing to them. For the buzzing noise of a static machine, or the peculiar hum of an electrical vibrator are not without their charms, especially so if the suggestion of the knife has already been made.

There is another type of gall stone disease in which it is impossible to make any diagnosis. We know that there is some lesion in the same difficult upper right quadrant, but we cannot speak with any degree of accuracy concerning it, and an exploration is all that we can advise. In no case of gall stone disease where we are reasonably sure of the diagnosis should we wait for the occurrence of chills, fever and jaundice before advising operation, for we would only be inviting danger to those we are called upon to protect.

After we are confidently sure of the existence of gall stones we cannot always be positive of their location, for sometimes stones remain in the common duct a long time without any sign of obstruction, and on the other hand, some of the worst cases of jaundice are seen where stones are only in the gall bladder and result in septic cholangeitis. As soon as we are convinced of our diagnosis, surgical treatment should be advised, provided there are no serious contradictions. The brilliant work of the Mayos along this line is more than convincing that surgery is the rational treatment, and furthermore, their results demonstrate that the end justifies the means.

Debate and contention among surgeons as to what is the proper procedure in surgical work on the gall bladder and its ducts is as yet in an unfinished state. Twenty years ago cholecystostomy was the usual routine. A little later cholecyst-interostomy was much the practice, then came the ideal cholecystotomy and now the almost universal practice of cholecystectomy is the rage. The results where stones are confined to the gall bladder alone are as gratifying as one could wish for. But the further a stone is removed from the gall bladder in the cystic or common duct, the more complicated and difficult is its removal, and it is from this group of patients that the highest mortuary statistics come.

Mayo says: "Looked at from a standpoint of mortality, cholecystostomy is the safest operation for the average case and must be considered the normal operation. In a series of fifteen hundred cases, in only one did stone re-occur in the gall bladder, and he does not think this a valid objection for leaving it in situ." Cholecystectomy has its place in such cases as early malignancy confined to the walls of the bladder, markedly dilated or contracted gall bladder, and in cases of phlegmonous or gangrenous cholecytitis. But as a routine practice it is bad for the following reasons:

It precludes the future operation of cholecyst-interostomy, should the common duct become contracted or obstructed by stone or adhesions and removes the ideal channel through which it is so easy to establish biliary drainage. Furehermore, usually one attempt to do any kind of operation on the bile passages after the bladder has been removed is sufficient to convince that it is the only safe and sure guide in locating the bile ducts.

The highest mortality comes from stone in the common duct, especially high when near the diverticulum of Vater or that part of duct included in the wall of the duodenum. Choledochotomy is the ideal operation for common duct stone, but since it is difficult to close up the incised duct so as to prevent leakage, a safer practice is to do choledochostomy. When that portion of the common duct in the wall of the duodenum has to be opened the compound and complicated operation duodeno-choledochostomy has to be done. This operation having been devised and first successfully performed with recovery of both stone and patient by McBurney in 1891.

In any operation whatsoever upon the bile passages, drainage is the factor that must not be lost sight of. Consensus of opinion now seems to be that if a patient is markedly jaundiced or in a condition of cholemia, to divide the operation into two sections, the first being purely for drainage and the second to relieve whatever patological condition that may have produced it. Lillehthal in a recent paper on biliary drainage gives the following conclusions: (1st.) That the scientific and judicious employment of preliminary drainage in obstructed jaundice will probably lessen the dangers of such steps that may be necessary for a permanent cure. (2d.) That this drainage should form the sole object of the surgeon's work until the factor of cholemia has been eliminated. (3d.) That radical operations should in most chronic cases be postponed until hepatic ebgorgement and icterus no longer exist.

When we can succeed in having operations on the bile passages done during the hours of election and not delayed until the time of necessity demands, just so soon can better results be promised. It has been mortality and complications of delay that have placed the operation for appendicitis on a sound surgical basis, and the same arguments should apply with equal force to the early operations for gall stone diseases.

### REFERENCES.

W. T. Mayo, Annals of Surgery, Vol. XLIV No. 2.
Lilienthal, N. Y. Med. Journal, Vol. LXXXV, No. 20.
Hancock, Annals of Surgery, Vol. XLIII, No. 1.
Rouff, N. Y. Med. Journal, Vol. LXXXI, No. 24.
Graham, N. Y. Med. Journal, Vol. LXXXII, No. 24.
McArthur, Journal A. M. A., Dec. 9, 1905.
Douglas, Surgical Diseases of Abdomen.
Robson, British Medical Journal, Feb. 24, 1906.

